# Towards remote healthcare monitoring using accessible IoT technology: state-of-the-art, insights and experimental design

**DOI:** 10.1186/s12938-020-00825-9

**Published:** 2020-10-30

**Authors:** G. Coulby, A. Clear, O. Jones, F. Young, S. Stuart, A. Godfrey

**Affiliations:** 1 Department of Computer and Information Sciences, Faculty of Engineering and Environment, Northumbria University, Newcastle Upon Tyne, NE1 8ST UK; 2 Director of Research, Ryder Architecture, Newcastle Upon Tyne, NE1 3NN UK; 3 Department of Sport, Exercise and Rehabilitation, Faculty of Health and Life Sciences, Northumbria University, Newcastle Upon Tyne, NE1 8ST UK

**Keywords:** Cloud connectivity, Gait, Remote monitoring, Sensors, Wearables

## Abstract

Healthcare studies are moving toward individualised measurement. There is need to move beyond supervised assessments in the laboratory/clinic. Longitudinal free-living assessment can provide a wealth of information on patient pathology and habitual behaviour, but cost and complexity of equipment have typically been a barrier. Lack of supervised conditions within free-living assessment means there is need to augment these studies with environmental analysis to provide context to individual measurements. This paper reviews low-cost and accessible Internet of Things (IoT) technologies with the aim of informing biomedical engineers of possibilities, workflows and limitations they present. In doing so, we evidence their use within healthcare research through literature and experimentation. As hardware becomes more affordable and feature rich, the cost of data magnifies. This can be limiting for biomedical engineers exploring low-cost solutions as data costs can make IoT approaches unscalable. IoT technologies can be exploited by biomedical engineers, but more research is needed before these technologies can become commonplace for clinicians and healthcare practitioners. It is hoped that the insights provided by this paper will better equip biomedical engineers to lead and monitor multi-disciplinary research investigations.

## Background

The western world spends > 80% of their time indoors often exposed to poor indoor environmental conditions, which can be detrimental to health and wellbeing [1]. The use of passive sensors for measuring indoor environments and monitoring impact on quality of life has become prevalent in recent years [2–6]. Of particular importance is the need for monitoring systems to track individuals and how they respond to environmental changes [7]. By localising the measurement of environmental factors and augmenting with data from wearable technologies, healthcare researchers can better understand health and wellbeing through a more holistic and personalised approach [8].

There is a trend toward personalised medicine with measurements suited to an individual’s ailments or needs. Individualised measurements could better identify health biomarkers, while longitudinally assessing habitual behavioural patterns [9]. To conduct such assessments, healthcare researchers need to move beyond the laboratory, towards free-living assessment. This involves longitudinal assessment of patients in their habitual environments, which can produce increased variability of measurements that may provide better insights to distinguish between physiological conditions [10]. This is because habitual environments are unsupervised and expose patients to a range of obstacles and tasks of daily living [11]. While healthcare researchers have proposed deploying monitoring equipment of the individual at scale, this has largely been unfeasible due to cost and complexity [12]. Moreover, there is a requirement to monitor beyond the individual by capturing insights about the general environment and how the individual performs daily tasks, which may negatively impact underlying pathology [6, 13]. Use of low-cost sensor technology could facilitate this methodological shift in patient assessment [8, 12] and in particular wearable sensing [11], but in many cases, the technology still requires a great deal of researcher intervention [11, 14, 15].

Emergent sensor technology is changing the landscape of how buildings, environments and individuals are monitored. This is in part due to the increasing affordability and accessibility of sensor technology being driven by the Internet of Things (IoT), which is regarded as an extension of the internet and is comprised of billions of globally interconnected devices [16, 17]. As a disruptive technology, IoT has the potential to positively impact healthcare, but are subject to limitations such as ongoing rapid technological changes [18]. Yet, that limitation is driving increased accessibility and affordability of IoT technology. Furthermore, marketing of sensor and associated technology is shifting from electronic engineers and computer scientists to other professions (e.g. construction, agriculture, manufacturing and education) and with it, innovative solutions to facilitate sensor integration and deployment.

The aim of this paper is to provide a narrative review while surveying current state-of-the-art of accessible IoT sensor technologies. Here, we specifically examine low-cost technologies and investigate their use by providing examples for pragmatic insights to biomedical engineers. We present an overview of current low-cost devices and technical specifications to inform biomedical engineers about the possibilities, workflows and limitations presented by these technologies within healthcare applications. By doing this, it is hoped that biomedical engineers can better investigate ideas and develop proof of concepts to work more productively with electrical engineers, computer scientists and healthcare professionals when outlining and scoping work within modern multi-disciplinary studies. To place the review in context of current challenges for biomedical engineers, this paper will investigate by means of experimental work approaches for remote environmental and physiological monitoring. It is hoped that the findings of this experimental work will showcase low-cost IoT approaches with pragmatic considerations for future biomedical investigations.

## Low-cost sensor technology

Sensors are a prevalent driver of IoT technology and they serve a multitude of purposes, from measuring people or places to systems or things. Sensors can be used to measure air quality or motoric activity, the latter which can help identify symptoms of underlying medical conditions, e.g. Parkinson’s disease (PD) [19]. Those type of sensors have taken a variety of form factors, from environmental sensors that use printed conductive plastics that can accurately detect the concentration of carbon dioxide (CO_2_) in the air [20] to smart clothes that integrate tri-axial accelerometers directly into garments [21]. Key to these developments is the increasing technological advancements in microelectromechanical systems (MEMS) [22].

### Initial prototyping tools: MEMS sensors and bench testing

MEMS use micro-engineering to integrate circuits and microscopic mechanical components into silicone microchips [23]. In doing so, it is possible to create micro-scale sensors with a range of sensing capabilities. Table 1 highlights the versatility and potential for MEMS technology within healthcare research. Whilst some research focuses around use of MEMS sensors for specific healthcare applications, researchers are exploiting these technologies to create accessible sensor fusion ehealth monitoring systems. For example, studies [24–28] previously combined a range of low-cost sensors to create monitoring systems that were able to remotely measure a variety of health conditions. Alternatively, Rienzo et al*.* [29] adopted a different sensor-fusion approach to combine three sensors [electrocardiogram (ECG), photoplethysmogram (PPG) and seismocardiogram (SCG)] to simultaneously measure heart rate from 12 sensor nodes (each containing 3 sensors) that could be placed on different anatomical locations. In doing so, they were able to take 36 unique and individualised, high-frequency measurements of heart rate.

**Table 1:** Examples of MEMS sensor use for healthcare

Authors	Year	Healthcare application	Sensor ID	Sensor type
Alberto et al. [35]	2020	Heart rate	MAX30003^c^	Electrocardiogram (ECG)
Bakar et al. [24]	2020	Body temperature Heart rate	MAX30205^c^ SEN11574^d^	Temperature Electrocardiogram (ECG)
Rienzo et al. [29]	2020	Heart rate Pulse	MAX30003^c^ MAX30101^c^ LSM6DSM^e^	Electrocardiogram (ECG) Photoplethysmogram (PPG) Seismocardiogram (SCG)^a^
Al-Naggar et al. [25]	2019	Heart rate Pulse Body temperature	MAX30003^c^ AFE4490^f^ MAX30205^c^	Electrocardiogram (ECG) Pulse oximeter Temperature
Anisimov et al. [36]	2019	Heart rate	ADS1292R^f^ ADAS1000^f^ MAX30003^c^ AD8232^g^	Electrocardiogram (ECG)
Portaankorva [26]	2018	Heart rate Activity monitoring	MAX30003^c^ LASM6DSL^e^ LIS3MDL^e^	Electrocardiogram (ECG) Accelerometer/gyroscope Magnetometer
Yudhana et al. [37]	2018	Sign language detection	MPU6050^h^	Accelerometer/gyroscope
Anik et al. [38]	2017	Activity recognition	MPU6050^h^	Accelerometer/gyroscope
Dawson [39]	2017	Medical implant security	ADXL362^g^	Accelerometer/gyroscope
Fitriani et al. [40]	2017	Activity recognition	MPU6050^h^	Accelerometer/gyroscope
Kardos et al. [41]	2017	Gait analysis	MPU6050^h^	Accelerometer/gyroscope
Mohanraj and Keshore [27]	2017	Body temperature Pulse Heart rate Emotion detection	MAX30205^c^ SEN11574^d^ AD8232^g^ 101020052^i^	Temperature Photoplethysmogram (PPG) Electrocardiogram (ECG) Galvanic skin response
Mota et al. [42]	2017	Gait analysis	MPU6050^h^	Accelerometer/gyroscope
Shaji et al. [28]	2017	Body temperature Blood pressure Pulse Heart rate Fall detection	MAX30205^c^ HoneyWell 26PC^b^ SEN11574^d^ AD8232^g^ ADXL362^g^	Temperature Pressure Photoplethysmogram (PPG) Electrocardiogram (ECG) Galvanic skin response
Al-Dahan et al. [43]	2016	Fall detection	MPU6050^h^	Accelerometer/gyroscope
Kim et al. [44]	2015	Medical implant security	ADXL362^g^	Accelerometer/gyroscope
Lei et al. [45]	2015	Fall detection	MPU6050^h^	Accelerometer/gyroscope
Wang et al. [46]	2015	Gait analysis	MPU6050^h^	Accelerometer/gyroscope

^a^Seismocardiograph measurements were conducted using a MEMS-based accelerometer/gyroscope

^b^HoneyWell have a range of 26PC sensors, but the authors have not declared the specific sensor used in their study

^c^Maxin integrated products

^d^SparkFun

^e^STMicroelectronics

^f^Texas instruments

^g^Analog devices

^h^TDK InvenSense

^i^Seeed studio

**Fig. 1:**
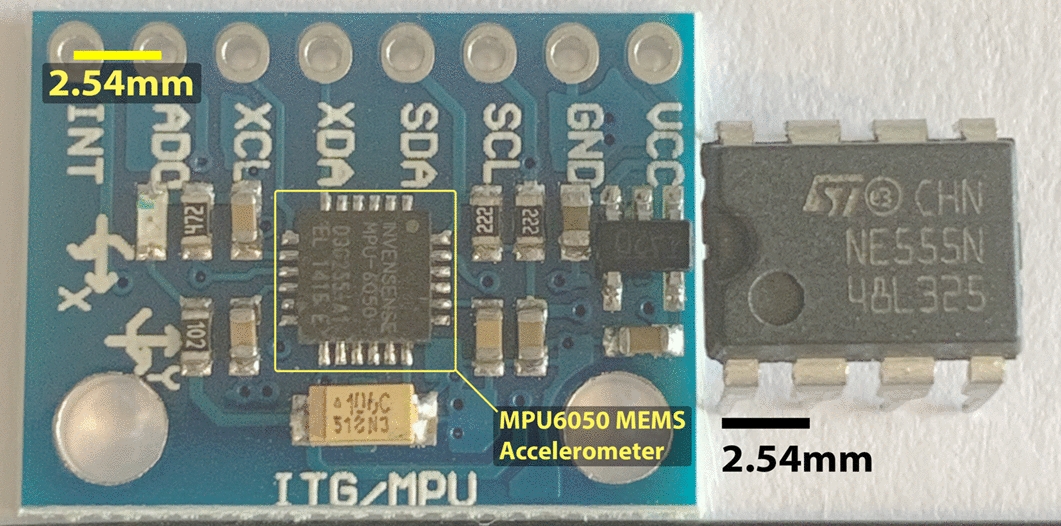
Scale of MEMS sensor breakout board, compared to a 555 timer chip with 2.54 mm pitch

One of the most prominent resources available for rapid prototyping electronic circuits are solderless breadboards, which is a device made of interconnected rows and columns designed to temporarily connect circuits. Typically, there are four rows of sockets on a breadboard, which are connected horizontally and are used for supplying power. The remaining sockets are connected vertically and are used for connecting components. The sockets are designed so that components and wires slot in, without needing to solder a permanent connection. Solderless breadboards are a mature approach for prototyping, so component manufacturers typically conform to the width and spacing of sockets when designing hardware. Therefore, by convention, many electronic components are standardised to have a pin spacing (known as pitch) of 2.54 mm [30]. This often makes MEMS sensors alone unsuitable for prototyping as they have a much smaller pitch, which vary from sensor to sensor. Sensors (e.g. Table 1) are often integrated onto ‘breakout boards’, which are small Printed Circuited Boards (PCBs) useful for prototyping and facilitate access to the pins on a microchip [31] by conforming to the 2.54 mm convention, Fig. 1. Many breakout boards can be used with little to no knowledge about electronic engineering. This is because much of the additional circuitry required to operate a MEMS chip is provided on the breakout board (Fig. 1), often exposing only inputs, outputs and voltage control pins. This is the reason why the number of pins on the MEMS component differs from the number of pins on the breakout board.

#### Ensuring fit-for-purpose monitoring

Sensor use within healthcare research is becoming more prevalent, but it has often been reactive rather than proactive as innovation in this field can be quite fractious [32, 33]. With continued uptake of emergent technologies, biomedical engineers must perform robust and vigorous bench testing (e.g. via tools outlined in “Initial prototyping tools: MEMS sensors and bench testing” section) to ensure new sensor-based technologies are valid and fit-for-purpose [32]. There is no absolute standard regarding sensor selection, as choosing an appropriate sensor will depend on what the researcher needs to measure and the subsequent digital endpoint(s) that is/are sensitive to the pathology in question [34]. Once fit-for-purpose sensors have been selected, appropriate and equally fit-for-purpose processing units (i.e. what the sensors are integrated into) must also be selected, to send control signals to the sensors as well as read and process sensor data.

## Communication and control: hardware

There are a variety of ways to communicate and control sensor technology, which can vary depending on the stage of production, requirements of the hardware or accessibility.

### FPGA/ASIC

For applications that require a great deal of power efficiency, whilst executing control algorithms in parallel and at high speeds, an Application-Specific Integrated Circuit (ASIC) may be required [47]. ASICs are microchips that contain an integrated circuit that is designed for a single application and cannot be reprogrammed [48]. This makes them suited to production level devices that do not need to change throughout the device’s lifecycle. Alternatively, Field Programmable Gate Arrays (FPGA) are reprogrammable. FPGAs are similar to ASICs as they contain integrated hardware circuits and once programmed can perform any logical function [49]. However, FPGA architecture differs from an ASIC and is comprised of an array of inputs and outputs (I/Os), logic blocks, interconnects and connection lanes. These interconnects can be programmed so that the connection lanes bridge a connection between I/Os and a series of logic blocks to form a circuit of components that are suited to a specific application [50].

Since FPGAs and ASICs require the configuration of hardware circuits, they have a steep learning curve and may lack general accessibility to those without circuit design experience. However, it is also possible to interface with sensors using a programmable Central Processing Unit (CPU), which is used for controlling hardware and software [51]. Within IoT applications, CPUs are typically integrated into a Microprocessor unit (MPU) or into a Microcontroller unit (MCU) which combines CPU with memory. That enables CPUs programming to execute processes whilst being able to read and write data during an execution [52]. The key distinction between MPUs and MCUs, is the latter combines the CPU and memory onto a single microchip making it act as a single-chip-computer, capable of executing programmed instructions [53].

### CPU

In contrast to FPGAs or ASICs, CPUs process algorithms in series, meaning they are not capable of running concurrent tasks. This can be overcome using multi-core CPUs, which combine multiple CPUs cores into a single processing unit, where each core can concurrently execute commands in series [54]. Another key distinction between CPUs and FPGAs is that whilst both architectures can be programmed, the program used in an FPGA is used to define how the hardware is configured, whereas the CPU executes the code as a series of instructions [50]. Since processing on an FPGA is done using hardware this means they are capable of handling analogue or digital signals, whereas a CPU is only capable of processing digital information. While this may seem like a major limitation for healthcare applications, one of the benefits of MCUs is that they typically contain a bus of general purpose input/output (GPIO) pins, which allow the device to send or receive both analogue and digital information from peripheral devices such as sensors [55].

Since the underlying CPU is capable of processing digital information only, analogue signals must first be converted to a digital signal or vice-versa. This is done using either analogue-to-digital convertors (ADC) or digital-to-analogue convertors (DAC) depending on the direction of the signal. When considering MCUs for healthcare applications and analogue signal measurement, it is important to consider the performance of the ADC to ensure that the device has sufficient resolution to be fit-for-purpose. This largely comprises of a trade-off between the sample rate, measured in samples per second (sps) and the bit resolution of the ADC, which refers to the number of discrete digital values an analogue signal can be mapped to. The more the bit resolution of the converted signal is lowered, the more the degradation of information is increased. Moreover, as the sample rate is increased, the ADC needs to convert a greater amount of information, which further reduces the bit resolution of the conversion [56]. Therefore, if biomedical engineers intend to take measurements from analogue sensors, at a high sample rate, it is important that they choose an MCU with an ADC that has a high bit resolution when operating at the desired sample rate. This is to ensure the quality of the digital signal that is converted from the analogue stream is of a high standard for accurate data capture and robust patient assessment.

### MCU

A key benefit of MCUs is their low-cost and accessibility, largely driven by open-source-based Arduinos—a range of inexpensive MCUs that are typically built onto development boards for rapid prototyping [57]. In software development, open-source code is typically distributed with a license that enables other developers to view, modify and share derivative works legally [58]. In much the same way, open-source hardware licenses allow the technology to be modified and distributed legally. This means that manufacturers and developers are free to clone, build, enhance and distribute hardware that builds upon the original infrastructure. Since derivative boards are based on the Arduino architecture, the way in which these microcontrollers are programmed has become standardised. The widespread adoption of these boards has not only incited rapid advancements in the capability of Arduinos, but it has also drastically reduced the costs of associated components.

Since their conception, Arduinos have taken a variety of forms and purposes. These include controllers for smart clothes that use inductive thread to control sensors to compact networked boards that are designed to interface with the IoT. For a full list of options and specifications, readers are directed to Arduinos product range,^1^ which outline the technical specifications of each board and categorises the boards according accessibility. Additionally, Nayyar and Puri [59] present a review of Arduino hardware, outlining the technical intricacies of each board. However, the Arduino product range is continuously evolving and many of the boards in that review have subsequently been discontinued, as is the nature of disruptive technology [18]. Whilst the details presented in Arduino’s product range provide detailed technical specifications, they lack aggregated information on the ADC/DAC capabilities of each device. To address this gap, Table 2 is provided to further guide biomedical engineers when choosing boards to suit the needs of their research projects.

**Table 2:** Arduino's product range, highlighting architectures and ADC/DAC capabilities

Board	Price^a^	Processor	Digital/PWM^b^	ADC bit resolution	ADC CHLs	ADC sample rate^c^ (ksps)	DAC bit resolution	DAC CHLs
Entry level								
UNO R3	$23	ATmega328P (8-bit)	14/6	10-bit	6	15	–	0
Nano	$21	ATmega328P (8-bit)	22/6	10-bit	8	15	–	0
Leonardo	$21	ATmega32U4(8-bit)	20/7	10-bit	12	15	–	0
Micro	$21	ATmega32U4 (8-bit)	20/7	10-bit	12	15	–	0
Nano every	$11	ATMega4809 (8-bit)	22/5	10-bit	8	115	–	0
Enhanced								
MKR zero	$26	SAMD21 (32-bit)	22/12	8/10/12-bit	7	350	10-bit	1
Zero	$43	SAMD21 (32-bit)	20/10	12-bit	6	350	10-bit	1
Due	$41	AT91SAM3X8E (32-bit)	54/12	12-bit	16	1000	12-bit	2
Mega 2560 Rev3	$41	ATmega2560 (8-bit)	54/15	10-bit	16	15	–	0
IoT								
Nano 33 IOT	$19	SAMD21 (32-bit)	14/11	8/10/12-bit	8	350	10-bit	1
Nano 33 BLE	$21	nRF52840 (32-bit)	14/14	12-bit	8	200	–	0
Nano 33 BLE sense	$32	nRF52840 (32-bit)	14/14	12-bit	8	200	–	0
MKR WAN 1300	$41	SAMD21 (32-bit)	8/12	8/10/12-bit	7	350	10-bit	1
MKR GSM 1400	$69	SAMD21 (32-bit)	8/13	8/10/12-bit	7	350	10-bit	1
MKR WiFi 1010	$33	SAMD21 (32-bit)	8/13	8/10/12-bit	7	350	10-bit	1
MKR NB 1500	$77	SAMD21 (32-bit)	8/13	8/10/12-bit	7	350	10-bit	1
MKR Vidor 4000^d^	$72	SAMD21 (32-bit)	8/13	8/10/12-bit	7	350	10-bit	1
MKR 1000	$37	SAMD21 (32-bit)	8/12	8/10/12-bit	7	350	10-bit	1
UNO WiFi Rev2	$45	ATMega4809 (8-bit)	14/5	10-bit	6	115	–	0

All information has been sourced from Arduino’s product range and the subsequent datasheets provided there

^a^Prices (as recorded on 10 July 2020) are rounded up to the nearest USD (ex. VAT)

^b^Pulse width modulation (PWM) is an emulated analogue signal created with high-frequency digital pulses

^c^ADC sample rates specified are in kilo-samples per second (ksps) and are achieved at the highest bit resolution of the ADC, lower bit resolutions can achieve sample rates greater than those specified above

^d^MKR Vidor 4000 has an on-board Intel^®^ Cyclone^®^ 10CL016 FPGA to supplement the SAMD21 MCU

Boards in Table 2 convert analogue signals to digital with at least a 10-bit resolution. Moreover, the sample rates of modern Arduinos enable them to be applicable for a range of healthcare applications as they exceed requirements for measuring high-frequency analogue signals, e.g. electrocardiographs [60]. As the technology continues to disrupt, modern Arduinos push the boundaries with new processors and higher resolution ADC capabilities. Furthermore, IoT is an increasing driver of technological development and Arduino’s own IoT range now come equipped with e.g. a range of wireless capabilities to suit a variety of remote measurement projects via Cloud services or MCU boards with built-in FPGA for additional programmable functionality. However, these come at an increased cost, inhibiting accessibility.

Derivative boards and inexpensive clone boards are an alternative, providing equal functionality much lower cost. For example, an official Arduino Uno R3 costs approx. $23 but a clone built to equal sizes and specifications is as little as $3.00. Although not supported by Arduino, clone boards will function the same as Arduino counterparts and will likely be compatible with Arduino software, as the latter supports third party manufacturers. However, biomedical engineers using clone boards should be aware that they would be unlikely to receive official support from Arduino for any clones.

The open-source nature of Arduino products means that derivative boards can also be created. Instead of aiming to create clones that offer equal functionality, derivative boards aim to extend the functionality of Arduinos by on-boarding features such as LCD screens, wireless communication and more powerful processors, which can be useful for providing real-time feedback from sensor readings. One example which is gaining popularity [61] is the ESP32.^2^ The latter cannot be directly compared to an Arduino development board as it is regarded as a system on chip (SoC), meaning that it is an entire system on a single microchip. These chips are considered a market leader as they integrate WiFi, Bluetooth Low Energy (BLE), dual-core processing and sensors onto a single chip [62]. Moreover, these chips are now being integrated onto a wide range of development boards that offer similar accessibility as Arduino development boards but with increased functionality and lower costs. One reason why SoCs (and the development boards built upon them) have been so successful within IoT development is that the entire chip can be reconfigured at run time to operate at extremely low power, making them suitable controllers for battery-powered IoT devices [63]. Furthermore, the ESP32 chip has 18 multi-resolution ADC channels capable of running 200 ksps at 12-bit resolution and two 8-bit DAC channels, which makes the chip comparable to the Arduino Due—one of Arduino’s largest form-factor development boards. Of note, while the ESP32 has an 18 channel ADC, two of those channels are occupied by integrated temperature and hall-effect sensors that detect magnetic fields and the temperature of its chip [63]. This means that for applications that do not make use of these sensors, the ESP32 has only 16 usable ADC channels, though this is comparable to the Arduino’s Due and Mega 2560 boards.

Unlike FPGAs and ASICs, Arduinos and their derivative microcontrollers were designed to be accessible to beginners yet flexible to accommodate skilled developers [57]. This makes them ideal for those that may not possess the prerequisite knowledge of an e.g. electrical engineer but wish to gain insights into IoT hardware development or become more knowledgeable about possibilities and limitations of the hardware.

### Software

The scale of data across the healthcare sector has been increasing and is expected to continue increasing exponentially as healthcare professionals adopt IoT solutions [64]. As more information is stored into healthcare models, challenges around transmission and storage of those models increases in tandem. IoT adds further complexity to the issues of data scale as devices typically send a telemetry stream, which is continuous data ranging in frequencies from seconds to weeks. Therefore, frequency of data transmission has a direct impact on the level of storage and the type of system that is needed to manage the stream.

Devices like Arduino processors must be programmed with a specific set of commands telling it which pins to read and write to and what to do with the data. Hardware manufacturers (e.g. Arduino, Adafruit, SparkFun) provide searchable databases of open-source code libraries (often accompanied with setup tutorials) that can be accessed from a web browser or their proprietary software.^3^ Thus, biomedical engineers can be more informed about the steps involved and understand the possibilities and challenges the hardware presents through the support of those tutorials and documentation.

## Cloud connectivity

IoT workflows extend beyond the development of sensor technology by developing software that collects, stores and analyses data streams. Open-source IoT software platforms are also becoming a driving force of accessibility and innovation. These platforms are typically centred on providing a web-based dashboard and a database to collect and display data from IoT devices. Biomedical engineers should be aware there are more than 600 known IoT platforms [65] and, whilst the sector is largely dominated by large corporations such as Amazon, Google and Microsoft [66], IoT cloud platforms are continuing to expand and fragment with niche platforms designed for specific use cases [65]. These platforms are typically centred on providing a web-based dashboard and a database to collect and display data. Many of these platforms are complex and feature-rich, with a range of integration protocols that can directly interface with MCUs [67–69]. However, many of these cloud platforms operate on a quota or a pay-as-you-go model, where users pay for services, storage or bandwidth they consume [70]. For IoT applications such as smart homes, this can be an affordable option as the frequency of events (*when an IoT device uses some of the quota*) can be sporadic or low-frequency; e.g. when a light turns on or off. In healthcare research, the frequency of data transmission may often need to be much greater, in the region of hundreds or thousands of samples/second. This currently creates multiple technical obstacles that make cloud-based remote monitoring of patients challenging.

### Rate limiting and transactional cost

When transmitting high-frequency sensor data to the cloud, a large volume of data can be accumulated in a short space of time. This will require large amounts of cloud storage and may require a great deal of bandwidth. Before adopting a cloud solution, biomedical engineers must be aware of how a user is charged for data, with regards to both storage and bandwidth. Given the number of available cloud solutions, a complete breakdown of costs involved with each service is beyond the scope of this paper. Instead, we present indicative costs associated with different subscription models from the key providers, Microsoft Azure, Amazon’ Web Services (AWS) and Google’s cloud platform (GCP) [66].

To demonstrate the speed in which message quotas would be consumed using cloud platforms, we extracted several ten second samples of raw tri-axial data from a low-cost commercial MEMS-based wearable accelerometer (AX3, Axivity, Newcastle, UK) in CSV format with timestamp information included. The sample rate was set at 100 sps (100 Hz) and so each sample contained 1000 rows (100 Hz × 10 s) of values. The average file size of the CSV data was approximately 33 kilobytes (KB). This file size was then used to compare the pricing for the three major cloud IoT platforms.

#### Microsoft Azure IoT Hub

Microsoft Azure’s IoT Hub has a range of pricing options and quotas (Table 3). Users of the service are billed monthly and charged according to the number of messages/day. For device-to-cloud messaging, the maximum of a single message equals 256 KB [71], meaning no single device can send more than that at any one time. However, that message size is far greater than the meter size for each tier, which is capped at a maximum of 4 KB for paid tiers and 0.5 KB for the free tier, Table 3. Therefore, while a single 256 KB message can be sent from an IoT device to the cloud, this message is segmented into 0.5 KB/4 KB segments and charged accordingly. Thus, a 256 KB message will expend 64 messages from the daily quota on paid tiers and 512 messages from the daily quota on the free tier. For high-frequency data, this quota can be quickly consumed. Using the example set out in “Rate limiting and transactional cost” section, a 67.1 KB message would consume 9 messages from paid tier subscriptions and 66 messages from free tier subscripts. At that rate, to monitor tri-axial data, values at around 100sps (100 Hz) for 24-h, approximately 71,280 messages would be consumed on a paid tier subscription. This would mean either the S1 or the B1 tier would be applicable. However, the daily message quota on free tier subscription would be completely consumed in around 20 min.

**Table 3:** Example of IoT hub pricing tiers

	Tier	Monthly cost	Messages/day	Meter size (KB)
Azure	Free tier	$0	8000	0.5
	Basic tier 1 (B1)	$10	400,000	4
	Basic tier 2 (B2)	$50	6,000,000	4
	Basic tier 3 (B3)	$500	300,000,000	4
	Standard tier 1 (S1)	$25	400,000	4
	Standard tier 2 (S2)	$250	6,000,000	4
	Standard tier 3 (S3)	$2500	300,000,000	4

	Monthly messages	Price^a^	Meter size (KB)	Connection cost^b^
AWS	< 1 billion	$1	5	$0.08
	1–5 billion	$0.80	5	$0.08
	More than 5 billion	$0.70	5	$0.08

	Data usage	Price/MB	Minimum charge (bytes)	
GCP	Up to 250 MB	$0.00	1024	
	250 MB to 250 GB	$0.0045	1024	
	250 GB to 5 TB	$0.0020	1024	
	5 TB and above	$0.00045	1024	

Data relating to tiers, pricing and message quotas was obtained from the pricing pages of Microsoft Azure [72], Amazon Web Services [73] and Google cloud platform [74] on 17 July 2020

^a^Per million messages

^b^Per million minutes

#### Amazon Web Services (AWS)

Similar to Azure, AWS IoT Core service involves chunking large messages and charging according with a maximum message size of 128 KB and a 5 KB meter size. Yet, unlike Azure, AWS tiers decrease in price as more messages are transmitted. If 10 s of tri-axial accelerometer recordings creates 33 KB of data, AWS would bill for 57,204 messages in 24-h. This equates to 1,710,720 messages over a 30-day period, where each million messages will be billed at $0.80—equalling $1.37 per month. Additionally, AWS also charge $0.08 per million minutes of connection, but for a single device, the price change is negligible as a device connected continuously for 30 days would cost $0.003456.

#### Google cloud platform (GCP)

GCP adopts a different quota system to Azure and AWS, instead charging according to the total amount of data transmitted rather than the total number of messages (Table 3). Additionally, instead of charging in data segments according to a meter size, GCP adopt a minimum charge approach when billing for transactions. Consequently, GCP encourage users to send fewer large messages rather than many small messages (unlike Azure and AWS). If 33 KB of tri-axial accelerometer data were sent from a device to GCP, instead of it being segmented and metered, prices would be calculated per megabyte (MB). In this instance, continuous data for 30 days would equate to 8.55 Gigabytes (GB) of data, costing $0.0045/MB. Therefore, total cost (*including first 250 MB free*) for 30 days would be $37.37. It is important to note that this cost only considers data being sent from the sensor, as GCP also have costs associated to the communication protocols used to send data. It is important for biomedical engineers to understand which protocols are available on a chosen platform as they can significantly impact the cost of data transmission.

### Communication protocols

Many cloud platforms accept a range of communication protocols, with two of the most popular protocols used within IoT platforms are Hyper Text Transfer Protocol (HTTP) and Message Queuing Telemetry Transport (MQTT). HTTP is a mature protocol for requesting and received data over the internet [75]. Within IoT, devices can send data over HTTP by attaching the data (known as payload) to the HTTP request being sent to a server. When the server receives the request, it returns a response to indicate the success or failure of the request/response lifecycle [76]. However, each request requires authentication and once the request/response lifecycle is completed the connection to the server is then closed [77]. This uses a lot of bandwidth and creates overheads for IoT devices that need to send high-frequency data to the Cloud. Contrastingly, instead of using a request/response lifecycle, MQTT protocol uses a publish/subscribe approach, where data are published to a server (message broker) and made available for subscription [76]. For example, an IoT device can publish a sensor reading to the broker and an IoT application (subscribed to the broker) can receive that data. A key benefit of MQTT over HTTP, for IoT applications, is that a persistent connection can be made to a broker, which allows devices to send multiple data payloads with a single authentication [75].

The fundamental differences between HTTP and MQTT have a substantial impact on cost within GCP. This is because GCP charges for each connection. For MQTT, monthly costs depend on how long the connection from a device is kept active. For example, if each device refreshes the connection every 15 min, 96 daily requests will be made to broker. Yet, whilst each request will be extremely small, GCP’s minimum charge means that every request is charged at 1024 bytes (1 KB), which equates to approx. 3 MB/month. Alternatively, HTTP makes a request and response every time data is sent. If 33 KB of data were transmitted every 10 s, 8640 messages would be sent daily. Since GCP would apply the minimum charge of 1 KB to each response, the HTTP responses alone would use the entirety of the 250 MB free quota. For this reason, in contrast to AWS and Azure, it would be important to send considerably larger amounts of data and to transmit less often when using GCP.

## Serial processing

Whichever cloud platform is adopted for an IoT solution, technological inadequacies of processing units can be a limitation when attempting to collect, store and transmit high-frequency data. As discussed previously, MCUs process data in series, meaning they execute each command one after another. Therefore, single core processors are unable to initiate the next command until the previous one is complete. On a single-core MCU, data transmission must, therefore, interrupt the data collection and the MCU will be unable to read sensor data until the data has been transmitted. This could also involve waiting for a response if transmitting over HTTP. Since it would be problematic to transmit every reading from a sensor running at a frequency of 100 Hz (100 sps), the MCU must read data from the sensor, perform analogue-to-digital conversion (if required), and store that data in memory. This whole process must also be executed within 10 ms (ms) to maintain a sample rate of 100 sps. When enough data has been collected in memory, the MCU must then send the data to the cloud. However, this instruction must also be executed within one of the 10 ms windows allocated to data collection, otherwise, the sample rate will drop. This problem could be mitigated using multi-core MCUs such as the ESP32, or devices that combine FPGAs with MCUs such as the MKR Vidor 4000. These devices would allow an uninterrupted data stream to be collected and stored, while simultaneously transmitting the data to the Cloud.

## Experimental case study: towards holistic IoT-based remote monitoring

From the plethora of IoT technologies that have been covered within our investigations, we conduct experimental work to investigate how current approaches could be undertaken by biomedical engineers for remote environmental and physiological monitoring. Here, we investigate a low-cost IoT approach for where an individual could be holistically monitored in their home with a focus on gait/walking assessment. The latter is commonly referred to the sixth vital sign [78] and has grown in considerable interest due to its ability to provide pragmatic insights to neurological conditions e.g. PD [79]. In brief, a conceptual model of gait suggests that numerous spatial and temporal characteristics (e.g. step length, step time variability) have clinical utility to examine onset and progression of PD [80]. This is important as a gait examination conducted under observation in the clinic can be used to diagnose, treat and manage those with PD. Traditionally, gait assessment in the clinic has proven useful but remains limited as the environment may not reflect daily life (e.g. good lighting, no obstacles) and those being assessed will perform the test optimally due to being observed [10]. Advances in wearable technology have created a methodological shift to quantify spatial and temporal gait characteristics beyond the clinic. It is hypothesised that these free-living characteristics can provide more insight due to the habitual manner in which they are generated. To date, evidence shows that there are differences in habitual gait compared to the clinic [11] with notable insights to fall risk assessment during prolonged assessment of those with PD [81].

Current state-of-the-art in longitudinal remote gait assessment predominantly aligns to placing an inertial-based wearable (typically tri-axial accelerometer) on the lower back for extended periods (up to 7-days when also considering ambulatory behaviours). Upon completion of recording, the wearable is collected in person or returned to the researcher by post. This is extremely inefficient, costly and may often result in damage (or loss) of wearables (and data). Furthermore, recent impact of the 2020 COVID pandemic brought clinical and research studies in this field to a halt due to isolating requirements for those with health conditions. Thus, there is a need to investigate how future habitual gait assessment could be best facilitated and maintained through the use of IoT technologies. Moreover, the addition of environmental information could augment gait assessment data, by providing healthcare professionals with greater insight into an individual’s living conditions (e.g. light quality of room) and how that may impact gait performance [12].

### Physiological measurement of gait: current state-of-the-art

Given fabrication of modern inertial-based wearables due to MEMS technology, they can generally be worn on any anatomical location but placement on the lower back conforms to harmonisation of two principal algorithms for gait quantification [82, 83] to generate 14 spatial and temporal characteristics [84] of clinical utility [85]. In brief, use of the continuous wavelet transform helps identify timings of the initial (heel strike) and final contact (toe off) for each step from the vertical acceleration of MEMS-based wearables, such as the AX3. The AX3 has been widely used for validated gait analysis studies in various clinical cohorts [86–89]. Those contact times coupled with the inverted pendulum model [90], which estimates change in height of the wearable due to attachment near the wearers’ centre of mass, provide pragmatic gait characteristics. Furthermore, identifying periods of gait (bouts of walking) from longitudinal assessment is feasible from a heuristic approach of (i) wearable location (accelerometer orientation) and (ii) recognising periods of interest from combined tri-axial inertial signals to define when the wearer is upright (mean accelerometer output) and moving (threshold to standard deviation). Once those periods of interest are located, they are analysed for initial and final contacts to deduce that the wearer is walking [91].

Previously, it was shown that accessible IoT-based technology (smartphone, inertial wearable and Raspberry Pi) could be used beyond the clinic to gather robust gait data under observation when compared to routine procedures of analysing, via manual data download and processing through Matlab^®^ based gait algorithms [92]. Although the latter platform is being used less by data scientists, it remains popular due to its extensive toolboxes and formally arranged documentation and so may be perceived as the standard reference for processing sensor data. Nevertheless, more popular approaches involving use of Python or Octave have been shown to be comparable to Matlab^®^ for gait characteristic analysis [14, 92].

### Exploring IoT approaches to remote assessment

When experimenting with the IoT and algorithm deployment, biomedical engineers may seek methods that are a continuum of existing and validated approaches. ThingSpeak™ is an open-source cloud platform built upon Matlab^®^ meaning it can run its code in the Cloud to perform real-time analysis and visualisations on incoming data streams from IoT devices.

Like many cloud platforms, ThingSpeak™ imposes rate limits and quotas and these could be a major limitation for longitudinal assessment and multi-patient monitoring. When transmitting data to ThingSpeak™, data can be sent as individual messages where one message could comprise a reading from up to eight sensors. Alternatively, those data can be batched and sent collectively (i.e. in bulk) but regardless of transmission method the rate cannot be greater than one every 15 s. Nonetheless, ThingSpeak™ limits the amount of readings that can be transmitted in a bulk update message, with free users being limited to 960 rows and paid subscriptions being limited to 14,400 [93]. Given a sample rate of 100 Hz, each 15 s period would consume 1500 messages, equating to 8,640,000 messages/day. ThingSpeak™ charges in units where each unit includes a quota of data channels and messages. For academic subscriptions, costing $250/unit, a single unit has a message quota of 33 million messages. Therefore, a single unit would last just under 4 days if data were continuously transmitted. For longitudinal and/or multi-patient monitoring, these costs could grow exponentially. However, if the platform were used to analyse snapshots of data, biomedical engineers could fine tune their algorithms throughout a study and monitor the progress without waiting until the end of the sampling period. For environmental monitoring, high-frequency transmission is not always necessary, so these limits are not a factor.

### Experimental setup and equipment

To test the feasibility of ThingSpeak™, we conducted an experimental investigation to compare Matlab^®^ and ThingSpeak™, within the context of gait analysis. For the purposes of our experimental investigation, we present AX3 data from a single user in their habitual setting. The participant wore a single AX3 (100 Hz, ± 8 g) on the lower back for 1-h during which time they were free to perform their normal activities. Ethical consent was granted by the Northumbria University Research Ethics Committee (REF: 16,335/335) and the participant gave informed written consent before participating in this study.

Since the AX3 lacks wireless connectivity, the device was plugged into a desktop computer and the data were extracted and exported to CSV format. These data were then analysed in Matlab^®^ using a usual approach and validated algorithm [91]. Subsequently, a Matlab^®^ analysis application was created on ThingSpeak™ that contained the same code as on the desktop. The CSV file was then imported into ThingSpeak™ and analysis of these data was performed in the Cloud.

**Fig. 2:**
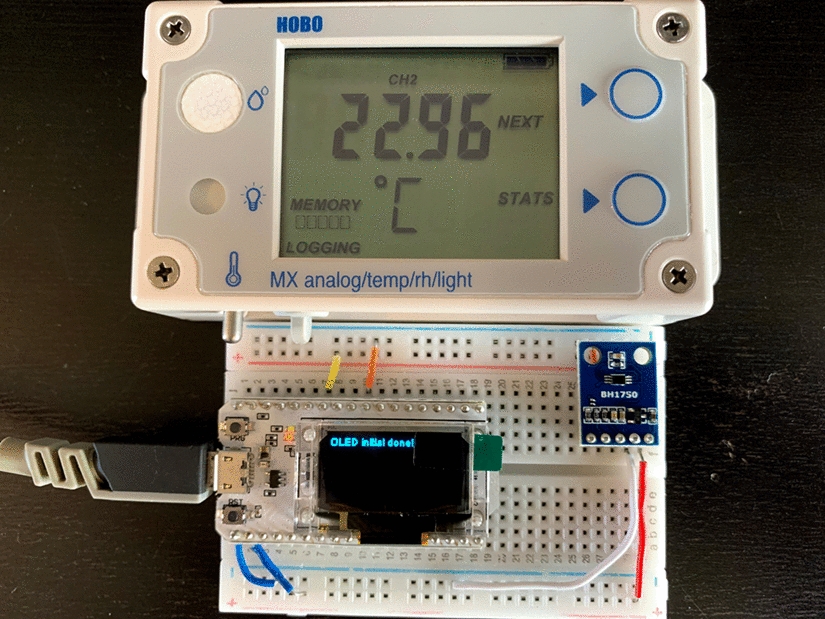
MX1101 light-intensity data logger and BH1750 ambient light sensor connected to ESP32 development board

Whilst the ability to run Matlab^®^ code in the Cloud is one of the primary benefits of ThingSpeak™, the platform also provides supported integration and code libraries for Arduino-based devices. Therefore, to test the potential of using the platform as a way of augmenting wearable sensor data with environmental data, we used a low-cost MEMS-based light-intensity sensor (BH1750) to collect and transmit data to ThingSpeak™ every 5 min using a Heltec ESP32 Wi-Fi 32 development board (Fig. 2). The frequency of data transmission was set to match a reference device, the HOBO MX1101 light-intensity data logger, which was simultaneously logging data on local storage to validate data from the BH1750. Data were captured from both devices consecutively for 5 days.

### Findings

The official Arduino support from ThingSpeak™ made connecting the BH1750 to the cloud a seamless process. Data were transmitted directly from the Heltec development board which was connected to the internet via Wi-Fi. Each time data were sent to ThingSpeak™, live graphs were updated allowing data from the IoT device to be quickly visualised. During data collection, it was also feasible to download ad hoc as a CSV file and analysed directly in the Cloud. Data transmission frequency meant there was no need to consider any rate limits imposed by ThingSpeak™ and the ESP32 was more than capable of transmitting the data at such a low frequency.

**Fig. 3:**
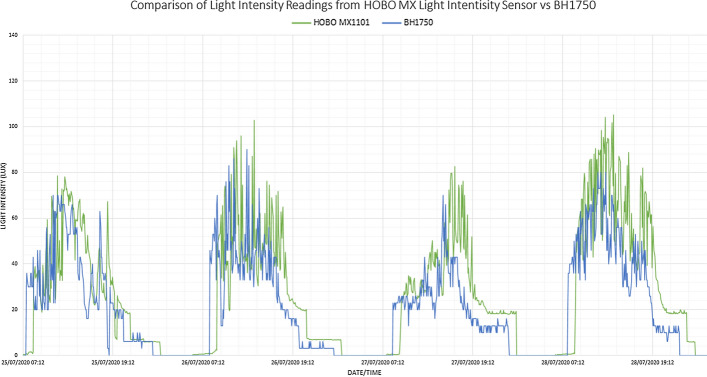
Data captured from HOBO MX1101 and BH1750

Regarding the data validation, the BH1750 was found to be highly correlative to the HOBO MX1101 sensor, with a Pearson correlation of 0.799. Moreover, Fig. 3 shows that whilst the accuracy of the BH1750 is slightly lower than the MX1101, the BH1750 is more responsive to changes in light intensity. The results of this experiment highlight the potential low-cost MEMS light sensors have in measuring ambient light intensity. They also highlight the potential of cloud platforms such as ThingSpeak™ for remote monitoring of an individual’s environment, given the longitudinal deployment of light-intensity sensors could be used to augment data from MEMS-based inertial wearables.

#### Gait: high-frequency data

**Fig. 4:**
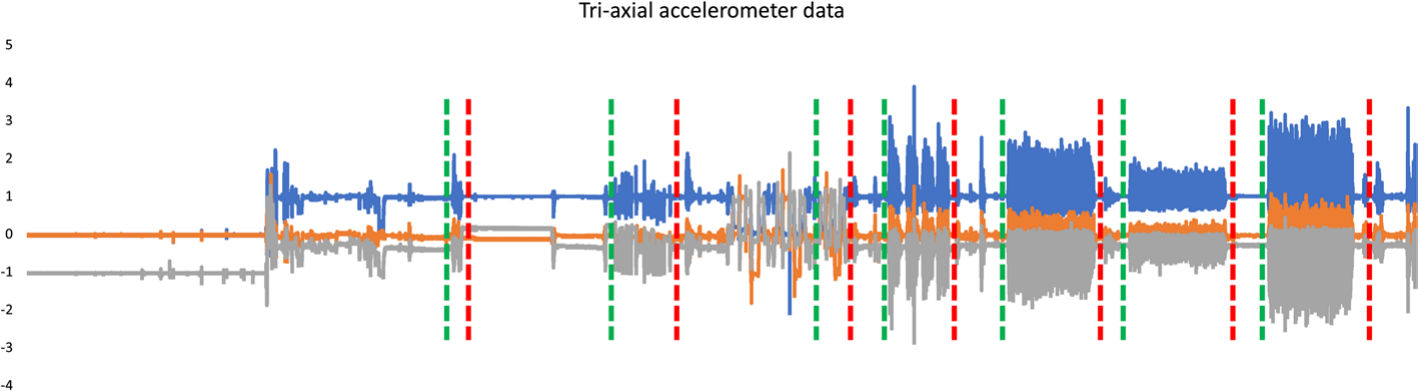
Free-living tri-axial accelerometer data (AX3). The vertical green and red indicate possible start/stop gait bouts

Individualised gait data were successful gathered and download via the usual desktop approach. The algorithm successfully segmented and identified gait events (Fig. 4, each bout was examined for initial and final contact times) and generated spatial and temporal outcomes (Table 4), presented previously [11, 81, 91]. In contrast, we found that while ThingSpeak™ could collect, store, visualise and analyse data from low-frequency environmental sensors, its ability to be used for existing gait assessment approaches within the IoT highlighted some major limitations. Although the rate limits imposed allow up to 14,400 readings to be sent every 15 s, it would appear that the platform is capable of processing high-frequency data akin to similar approaches via a desktop. However, during a bulk update, ThingSpeak™ checks no duplicate rows exist by comparing the timestamp of each reading. While this validation process accepts milliseconds and microseconds resolution timestamps, ThingSpeak™ rounds these to the nearest second, making it unsuitable for high-frequency data. Given the 1 Hz frequency limitation, to test how the Cloud-based Matlab^®^ Analysis compared with desktop approach, we circumvented the timestamp checks by changing timestamps to epochs in (seconds). This allowed high-frequency gait data upload for analysis.

**Table 4:** Individualised gait outcomes from all free-living data

Gait characteristics	Mean values across many bouts (s)
Step time	0.541
Stance time	0.711
Swing time	0.489
Step length	0.689
Step velocity	1.276

#### *Gait: analysis *via* IoT*

Reading data via a ThingSpeak™ channel instead of from a CSV file stored on a desktop uncovered further limitations. First, ThingSpeak™ limits readable data to 8000 rows, which meant that analysis had to be batched into 80 s sample windows. Once complete, a further error was encountered as the code utilised (e.g. filtering) functions from Matlab^®^ toolboxes that were not present in ThingSpeak™. Despite the removal of filtering processes, further errors were encountered, which highlighted fundamental differences between the two computation engines. While attempts were made to evaluate the IoT approach to gait assessment, in its current state, ThingSpeak™ is currently unsuitable for collecting high-frequency biomedical research data.

## Discussion

This paper presented a narrative review and survey of current state-of-the-art for accessible and low-cost IoT sensor technology. In doing so, we presented pragmatic insights of current technologies and the technical specifications that could present opportunities or limitations to biomedical engineers. One of the key benefits to these technologies is their low cost, meaning it is feasible to create scalable sensor fusion devices that incorporate a range of sensors for monitoring patients. Moreover, such sensor fusion devices could enable biomedical engineers to augment wearable biomedical sensing devices with environmental sensors to provide more context to e.g. gait outcomes, which would help them move their research beyond the laboratory and into free-living conditions.

### IoT hardware

Advancements in MEMS technologies allow a range of sensing capabilities that can aid biomedical engineers. However, many of these devices deal with high-frequency analogue signals, which present a new set of challenges, which biomedical engineers must consider when specifying both the sensors and the processing units that will collect data from the sensor. For many healthcare applications, such as ECGs and electroencephalogram (EEGs), high-frequency sampling is a requirement [94]. For this reason, it may be necessary to exploit the technological capabilities of ASICs or FPGAs, which can capture multiple high-frequency analogue signals simultaneously. However, open-source microcontrollers such as Arduino have driven the industry to develop boards that are demonstrably capable within this field. Whilst microcontrollers were traditionally limited by being unable to execute tasks concurrently, multi-core microcontrollers are now becoming more prevalent. Moreover, whilst MCUs cannot process analogue signals directly, due to the limitations of the internal CPU, this paper has demonstrated how advancements in ADC/DAC technologies are enabling MCUs to perform continually higher resolution conversions of analogue signals at high frequencies. Yet, for these devices to be considered IoT devices, there is a need to connect these devices to the internet. Networked MCU development boards are becoming more prevalent, boasting a range of wireless connection options that enable these devices to not only collect and process sensor data, but also transmit these data to the cloud IoT platforms.

### Cloud computing

From the three major platforms, AWS was found to be the cheapest platform overall, especially when using many devices. Contrastingly, GCP was found to be significantly more expensive. Nevertheless, the unique pricing model adopted by Google, means that the platform is better suited for transmitting large amounts of data infrequently, as opposed to Azure and AWS, which favour regular small amounts. Whilst it would not be possible to evaluate all of the cloud platforms here, we identified ThingSpeak™, an open-source Cloud IoT platform built on Matlab^®^. It could be reasoned that ThingSpeak™ may be a logical next step for biomedical engineers who are well versed in Matlab^®^ and wishing to explore Cloud IoT platforms. However, it is important for biomedical engineers to perform more rigorous bench testing of emergent technologies to ensure they are fit-for-purpose. For this reason, we conducted an experimental case study, to explore the suitability of MEMS technologies, MCUs and the ThingSpeak™ platform, to ensure they met our expectations. We found that low-cost infrequent data collection is feasible using the ThingSpeak™, which make the platform suitable for environmental data collection. Currently, there are limitations that make the platform unsuitable for physiological monitoring, namely rate limiting that curtails data logging to 1 Hz which is unsuitable for e.g. spatio-temporal gait analysis. Given the current limitations of ThingSpeak™, biomedical engineers invested in Matlab^®^ will likely need to explore sending data from IoT sensors to a cloud storage platform with connections to Matlab^®^ software on a desktop. For those not invested in Matlab^®^, it seems more appropriate to explore Python for analysis, given that it is comparable to Matlab^®^ and available on all three major Cloud IoT platforms.

### Experimental work

We focused the experiment on gait analysis and the augmentation of environmental data, due to emergence of the former as a pragmatic patient monitoring outcome. Our evaluations found that current limitations with the ThingSpeak™ platform make the platform suitable for biomedical researchers, due to the inability to process high-frequency data. Moreover, whilst the platform claims to run Matlab^®^ code in the Cloud, the two computation engines result in differences in how the code runs, making it unusable for complex analyses. This is exacerbated by the fact that ThingSpeak™ has a limited toolbox in comparison to desktop-based Matlab^®^.

Our experiment did not focus on one of the fundamental issues of the current state-of-the-art in gait monitoring, which is the need to wait until the end of the sampling period before collecting data. However, this process could be streamlined by utilising smartphone interactions with Cloud computing that could facilitate optimal remote gait data capture. Biomedical engineers could then use Matlab^®^ to collect data from the Cloud and access the data published by the mobile device. However, such experimentation would be beyond the scope of the experimental work presented here.

Whilst ThingSpeak™ was identified as currently being unsuitable for gait assessment, it could be a useful and inexpensive way for biomedical engineers to augment environmental data with healthcare data to provide more context during habitual assessment. Our experiment highlighted that low-cost MEMS technology can provide valid data which can be suitably collected, analysed and visualised via ThingSpeak™.

### Future research

The technologies explored here could be used to improve the current workflows within gait analysis. If low-cost, open-source devices like the AX3 could be controlled by a networked device, such as an ESP32, biomedical engineers could send a message to the device and request a snapshot of data at a given time, whilst the device simultaneously collects and stores the longitudinal data locally. Since these sensors are inexpensive, it would be possible to create a sensor fusion device that incorporates accelerometers (and gyroscopes) with an e.g. Global Positioning System (GPS) to provide location and elevation data when patients leave their homes. Moreover, these devices could communicate with smart home devices, such as smart lights, cameras, or environmental sensors, to augment and provide context to free-living gait assessment.

## Conclusion

Given the pervasiveness of IoT technologies, healthcare will become more reliant on multi-disciplinary research teams to break-ground with these disruptive technologies. For this reason, it is important that biomedical engineers become familiarised with the core concepts of IoT technologies so that they can be better informed of the technological capabilities and the challenges these technologies present. In doing so, biomedical engineers will be better positioned to not only lead investigations, but also monitor the progress throughout.

## Data Availability

The datasets used in the experimental work are not publicly available. Though, the conclusions surrounding this experimentation were based on the analysis process, not the data itself, so the conclusion in this manuscript do not rely on these data specifically.

## Abbreviations

ADC: Analogue-to-digital convertor
ASIC: Application-Specific Integrated Circuit
AWS: Amazon Web Services
BLE: Bluetooth Low Energy
CO_2_: Carbon dioxide
CPU: Central Processing Unit
CSV: Comma separated values
DAC: Digital-to-analogue convertor
ECG: Electrocardiogram
EEG: Electroencephalogram
FPGA: Field Programmable Gate Arrays
GB: Gigabyte
GCP: Google cloud platform
GPIO: General purpose input/output
GPS: Global Positioning System
HTTP: Hyper Text Transfer Protocol
I/Os: Inputs and outputs
IoT: Internet of Things
KB: Kilobyte
ksps: Kilo-samples per second
MB: Megabyte
MCU: Microcontroller unit
MEMS: Microelectromechanical systems
MPU: Microprocessor unit
MQTT: Message Queuing Telemetry Transport
ms: Millisecond
PCB: Printed circuit board
PD: Parkinson’s disease
PPG: Photoplethysmogram
PWM: Pulse width modulation
SCG: Seismocardiogram
SoC: System on chip
Sps: Samples per second

## Footnotes

1 Arduino product range https://www.arduino.cc/en/main/products.

2 Espressif systems https://www.espressif.com/.

3 Readers are directed to the Arduino Code Library List [95], which is an automatically generated database of libraries. This list contains approx. 3000 libraries, which provide detailed license, author and version information as well as links to download source code.
